# Lymphedema Prevalence and Treatment Benefits in Cancer: Impact of a Therapeutic Intervention on Health Outcomes and Costs

**DOI:** 10.1371/journal.pone.0114597

**Published:** 2014-12-03

**Authors:** Kimberly M. Brayton, Alan T. Hirsch, Patricia J. O′Brien, Andrea Cheville, Pinar Karaca-Mandic, Stanley G. Rockson

**Affiliations:** 1 Stanford Center for Lymphatic and Venous Disorders, Division of Cardiovascular Medicine, Stanford University School of Medicine, Stanford, California, United States of America; 2 Cardiovascular Division, University of Minnesota Medical School, Minneapolis, MN, United States of America; 3 Hematology/Oncology Division, University of Vermont, Burlington, VT, United States of America; 4 Physical Medicine and Rehabilitation, Mayo Clinic, Rochester, MN, United States of America; 5 Division of Health Policy and Management, School of Public Health, University of Minnesota, Minneapolis, MN, United States of America; University of Colorado Denver, United States of America

## Abstract

**Background:**

Lymphedema is a common complication of cancer therapeutics; its prevalence, treatment outcomes, and costs have been poorly defined. The objective of this study was to examine lymphedema prevalence among cancer survivors and to characterize changes in clinical outcomes and costs associated with a defined therapeutic intervention (use of a pneumatic compression devices [PCD]) in a representative, privately insured population.

**Methods and Findings:**

Retrospective analysis of de-identified health claims data from a large national insurer for calendar years 2007 through 2013. Patients were required to have 12 months of continuous insurance coverage prior to PCD receipt (baseline), as well as a 12-month follow-up period. Analyses were performed for individuals with cancer-related lymphedema (n = 1,065). Lymphedema prevalence was calculated: number of patients with a lymphedema claim in a calendar year divided by total number of enrollees. The impact of PCD use was evaluated by comparing rates of a pre-specified set of health outcomes and costs for the 12 months before and after, respectively, PCD receipt. Lymphedema prevalence among cancer survivors increased from 0.95% in 2007 to 1.24% in 2013. PCD use was associated with decreases in rates of hospitalizations (45% to 32%, p<0.0001), outpatient hospital visits (95% to 90%, p<0.0001), cellulitis diagnoses (28% to 22%, p = 0.003), and physical therapy use (50% to 41%, p<0.0001). The average baseline health care costs were high ($53,422) but decreased in the year after PCD acquisition (−$11,833, p<0.0001).

**Conclusions:**

Lymphedema is a prevalent medical condition that is often a defining attribute of cancer survivorship. The problem is associated with high health care costs; Treatment (in this instance, use of PCD) is associated with significant decreases in adverse clinical outcomes and costs.

## Introduction

Lymphedema is a vascular disorder that, in the Western world, arises most commonly as a consequence of cancer or its treatment. This is the most prevalent form of acquired or “secondary” lymphedema [1], [2]. While several single-center cohort studies have reported estimates of the prevalence of cancer-related lymphedema among breast cancer patients [3], the existing literature fails to fully define the population-based prevalence, health outcomes, and treatment costs of this disorder [4].

The advent of lymphedema carries substantial clinical implications for the affected cancer survivors, implicating profound losses in physical and psychosocial functioning [5]–[8]. Insight into the etiology and natural history of lymphedema has improved, but there is no cure [9]. Thus, the failure to treat lymphedema is associated with major adverse clinical outcomes [10].

Several treatment alternatives can effectively reduce lymphedema symptomatology and severity. For example, specific exercises are known to enhance limb mobility [11]–[13]. Case series have demonstrated that physical interventions such as manual lymphatic massage, multilayer bandaging techniques, and application of compressive garments can effectively reduce tissue fluid volume [14]–[16]. Recently, prospectively acquired data have also confirmed the effectiveness of adjunctive pneumatic compression device (PCD) therapies in diminishing edema volume and in improving patient-reported symptoms [16]–[19].

The impact of lymphedema on health costs and the potential benefits of therapy have been inadequately characterized. Prior studies in breast-cancer populations have suggested that the development of lymphedema adds significantly to the costs of disease management [20]. However, prior investigations have evaluated neither the overall health care costs of lymphedema management, nor the impact of any available therapeutic intervention in a large, representative national population.

To address these knowledge gaps, we conducted a retrospective analysis of a large private insurance claims administrative database for calendar years 2007 through 2013. Claims data are increasingly recognized as a valuable resource, facilitating estimates of recognized disease prevalence during long durations of follow-up analysis [20]–[26]. The goals of the current investigation were: (1) to estimate, for the first time, population trends in lymphedema prevalence and outcomes in cancer; (2) to identify the association of PCD use (one of the available therapeutic interventions) with these clinical outcomes, and (3) to define the health care costs of lymphedema in the context of this form of therapeutic intervention. We distinguished those outcomes and costs that were lymphedema-related from the general outcomes and costs, based on claims coding. We used a pre/post study design and compared the rates of a pre-specified set of relevant health outcomes and costs for the 12 months before and after PCD receipt.

## Methods

The IRB of Stanford University waived the need for ethical approval for our study. All the administrative health claims data was received anonymously from a de-identified Normative Health Information (dNHI) database between 2007 and 2013 for this study. The database consists of proprietary de-identified administrative health claims data from Optum Insight Inc. (Eden Prairie, MN). The database was not directly accessed by the authors. Search protocols were defined and analysis was conducted by Optum Insight personnel at the direction of the authors. There is no website ink available to the database. Permission for use of the analytical results and data supporting those results was obtained from Louis Brooks Jr, Vice President, Data Technology and Marketing Analytics at OptumInsight.

### Setting and Data Source

De-identified administrative health claims data from the de-identified Normative Health Information (dNHI) database were accessed between 2007 and 2013 for this study. dNHI includes more than 34 million individuals each year, comprised of both commercially-insured and Medicare Managed Care enrollees from a large United States (US) national managed care health insurer affiliated with Optum, Inc. (Eden Prairie, MN). The enrollment database includes a geographically diverse US population (16% West, 20% Midwest, 36% South, and 27% Northeast). In addition, the age and gender distribution of the dNHI is similar to that reported by the US Census Bureau for the commercially insured and the Medicare Managed care population.

All the administrative health claims data was received anonymously from the dNHI database. The database consists of proprietary de-identified administrative health claims data from OptumInsight Inc. (Eden Prairie, MN). The database was not directly accessed by the authors. Search protocols were defined and analysis was conducted by OptumInsight personnel at the direction of the authors. There is no website ink available to the database.

The dNHI includes enrollment data, as well as medical and pharmacy claims data. Medical (facility and professional) claims include diagnosis codes recorded with International Classification of Disease, Ninth Edition, Clinical Modification (ICD-9-CM), procedures recorded with ICD-9-CM procedure codes, Current Procedural Terminology (CPT) codes, or Healthcare Common Procedure Coding System (HCPCS), and revenue codes.

No identifiable protected health information was accessed during this study and de-identified data were accessed in accordance with the Health Insurance Portability and Accountability Act. Therefore, institutional review board approval was not required for this study.

### Study Populations

**Figure 1:**
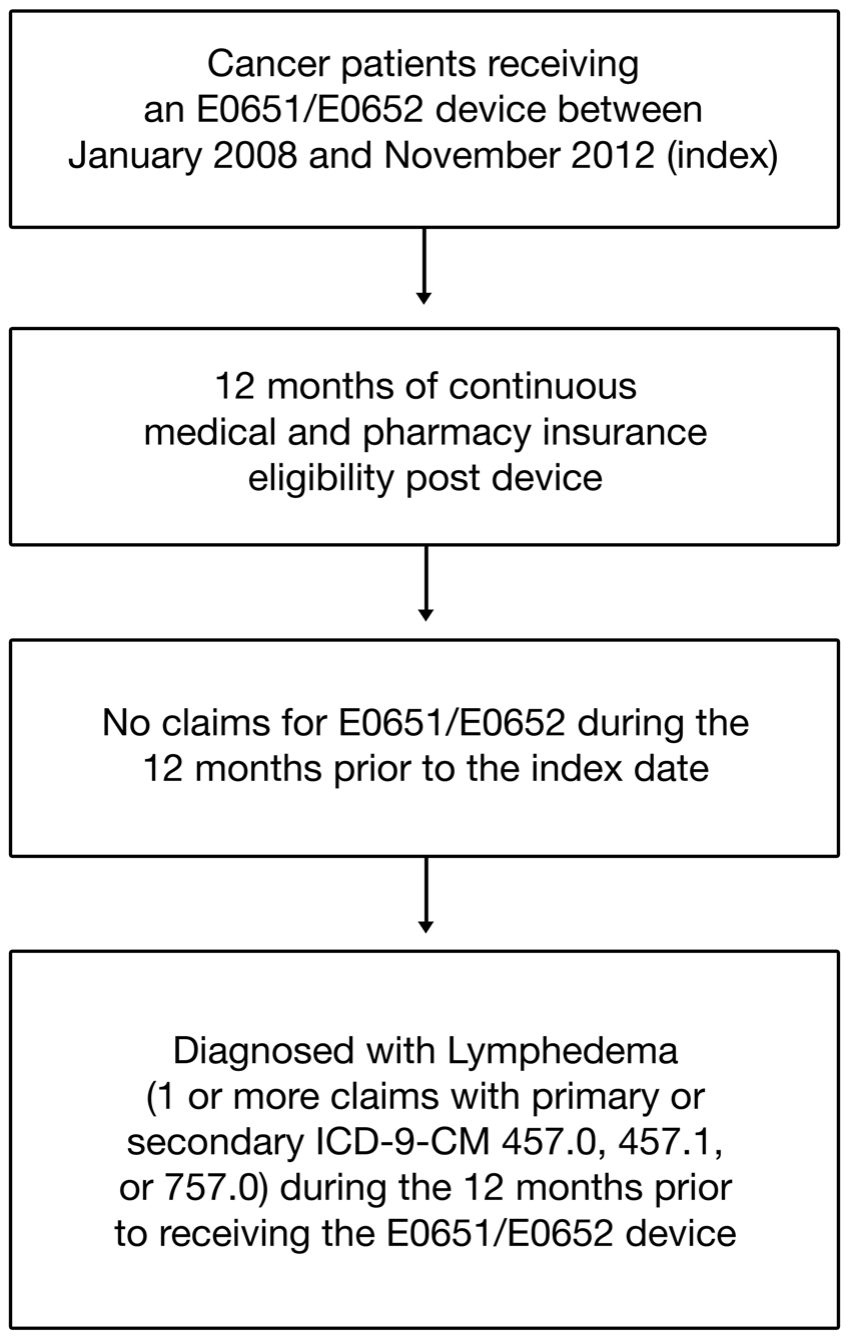
Flow diagram depicting the selection strategy for patients included in this analysis.

To estimate trends in lymphedema prevalence, we included all individuals in the analysis who had ≥1 day of medical benefit eligibility in the dNHI database during the timeframe of January 2007 through September 2013. We assigned patients to the cancer-related lymphedema cohort, based upon a primary cancer diagnosis, if they had one or more medical claims with ICD-9-CM diagnosis codes 140.xx-195.xx or 199.xx-209.xx (n = 950,033 in 2007; 1,005,372 in 2008; 1,078,822in 2009; 1,092,067 in 2010; 1,135,972 in 2011; 1,156,326in 2012; 1,188,860 in 2013). We classified individuals as having lymphedema if they had 1 or more medical claims with primary or secondary ICD-9-CM of 457.0, 457.1, or 757.0. Within the cancer cohort, in order to identify the association of PCD use with clinical outcomes and health care costs, we followed several steps of inclusion/exclusion (**Figure 1**). First, because we were interested in capturing pharmacy costs as well as medical costs, we required ≥1 day of pharmacy benefit eligibility during the year when the patient had medical benefit eligibility (resulting in an average exclusion of approximately 11 million individuals each year). Second, we restricted the sample to individuals who had a claim for a simple or advanced PCD identified with HCPCS codes E0651 (pneumatic compressor, segmental home model without calibrated gradient pressure) or E0652 (pneumatic compressor, segmental home model with calibrated gradient pressure) during the time period of January 1, 2008 through November 31, 2012 (n = 21,104). Third, we required the study sample to have at least 12 months of continuous medical and pharmacy insurance eligibility prior to receiving the PCD (n = 6,760). Fourth, because we wanted to focus on the first PCD, we excluded those with a claim for another PCD during the one year prior to receiving the PCD (n = 6,702). Fifth, because our primary interest was PCD users with a lymphedema diagnosis, and inasmuch as PCDs are also prescribed to treat other vascular diseases, such as chronic venous insufficiency and other diseases associated with limb edema, we further restricted the sample to individuals with at least one claim with a primary or secondary diagnosis code for lymphedema at any time during the 12 months prior to receiving the E0651/E0652 device (n = 3,415). Finally, we required individuals to have a primary cancer diagnosis in the baseline. We refer to this final study sample of 1,065 cancer patients as the *PCD study sample*.

### Patient Demographic and Clinical Characteristics

The dNHI database included information on patient demographic characteristics such as age and gender. Specific individual level socioeconomic status data elements, including race/ethnicity and household income category, can be linked to the dNHI. The data populating the socioeconomic status elements were available for approximately 75–85% of enrollees in the dNHI and are generated by a combination of self-report, modeling, census data and a variety of other individual-level and population-level sources.

In addition, claims data were used to identify co-morbid conditions other than cancer in the *PCD study sample* during the 12 months prior to receipt of the PCD (baseline). Patients were identified as having baseline obesity, diabetes, hypertension or renal disease based on the relevant ICD-9-CM and CPT/HCPCS codes in their medical claims. Finally, the Charlson co-morbidity score was calculated using the diagnosis and procedure codes during the 12 months prior to receiving the PCD [24].

### Lymphedema Prevalence

We calculated the annual prevalence of lymphedema in the dNHI database as the number of individuals with a lymphedema claim in a calendar year divided by the total number of individuals with ≥1 day of medical benefit eligibility and a primary cancer diagnosis in the dNHI database during that same calendar year.

### Clinical Outcomes and Health Care Costs

We separately considered a pre-specified set of relevant clinical outcomes for each patient in the *PCD study sample*, respectively, for the 12 months before PCD receipt and the 12 months after PCD receipt. These outcomes included hospitalizations, outpatient visits, episodes of cellulitis, and courses of lymphedema physical therapy. Episodes of cellulitis were identified as the number of medical claims with a primary or a secondary diagnosis code for cellulitis. Use of physical therapy was defined as having any medical claim with a CPT/HCPCS code for physical therapy. Courses of physical therapy were defined as physical therapy cycles separated by 15 days or more.

We used the American Medical Association place of service codes provided in claims to designate costs in various health care sites for each patient in the *PCD study sample*, separately, for the 12 months before and after PCD receipt. The settings included home health, emergency, hospital inpatient, hospital outpatient, and office visits, with separate aggregation of durable medical equipment, laboratory, and pharmacy expenses. In the analysis of outpatient costs, we distinguished physical therapy claims (claims which included a physical therapy CPT/HCPCS code) from any other service provided in the hospital outpatient setting.

Total costs were calculated as the sum of payment by the health plan and beneficiary, facility payments, and professional service fees. All clinical outcomes and costs were designated as lymphedema-related if the corresponding claim had a primary or secondary ICD-9-CM of 457.0, 457.1, or 757.0. These diagnosis codes were chosen to capture, as broadly as possible, the subgroup of patients who were assigned a specific lymphedema diagnosis. Lymphangitis (457.2) was excluded because it represents an acute inflammatory presentation that is not limited to the lymphedema population; swelling of limb (728.91) was excluded because of its excessively non-specific nature.

### Analysis

**Figure 2:**
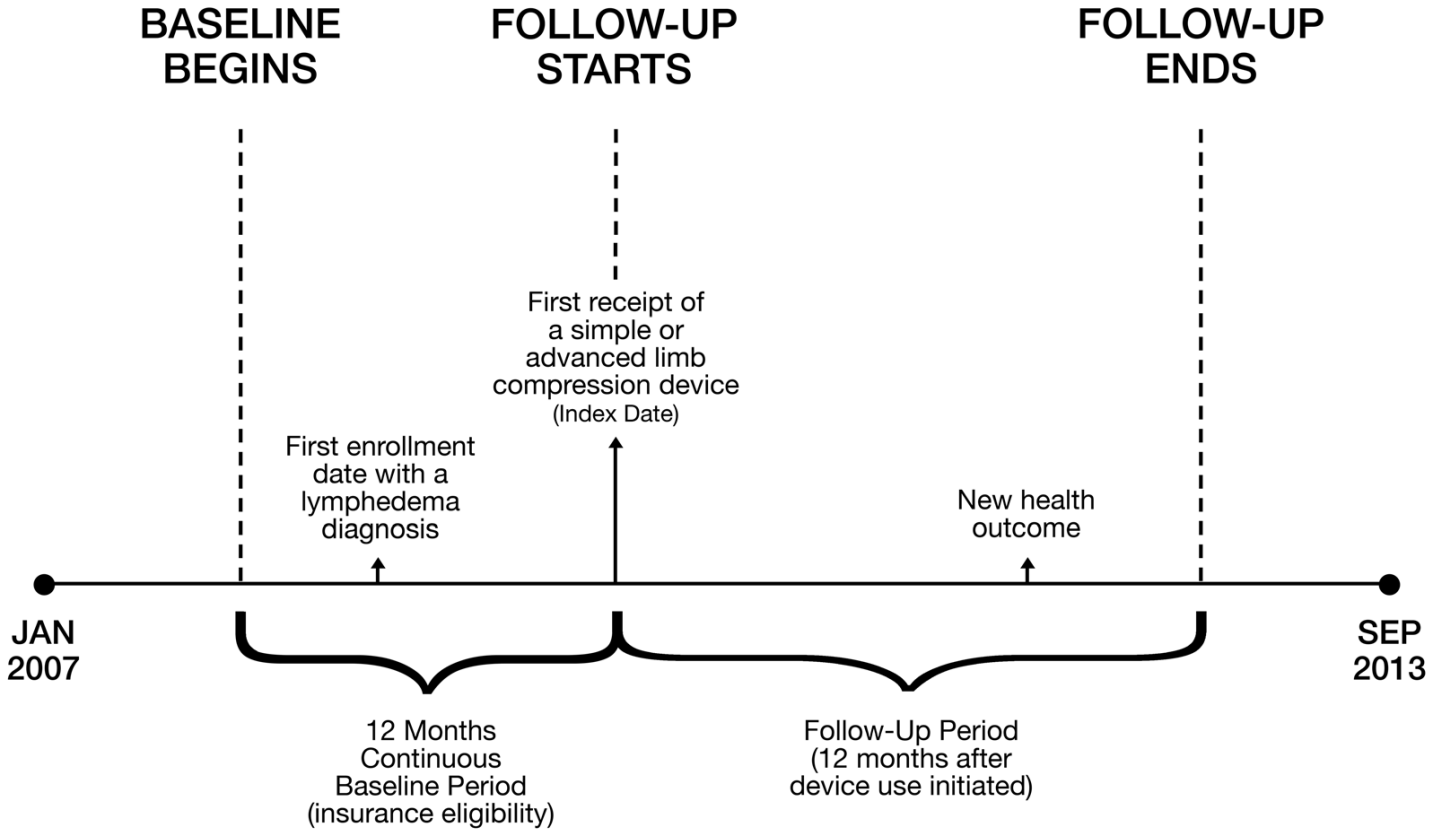
Schematic of the study design protocol.

The observation period for each individual patient was determined from a pre-specified “index date”, defined as the first claim date at which a simple or advanced PCD (HCPCS codes E0651 or E0652) was listed. The baseline period was defined as inclusive of data obtained during the 12 months prior to the index date. The primary observation period was comprised of the baseline period plus a 12-month follow-up period after the Index Date (**Figure 2**).

We compared the rates of the pre-specified set of relevant clinical outcomes and the health care costs per patient in each setting in the year before PCD receipt to the corresponding rates and costs in the year after PCD receipt.

Continuous variables were tested pre-PCD minus post-PCD with a paired t-test. Binary variables were tested with McNemar's test. Analyses were carried out with the SAS statistical package, version 9.2 (SAS Institute, Cary, North Carolina), with p<0.05 considered significant.

## Results

### Prevalence

In 2007, 9,025 of the 950,333 cancer patients in the dNHI database had lymphedema, reflecting a prevalence of 0.95%. The prevalence increased slowly during each subsequent year, rising to 0.977% (9,827/1,005,372) in 2008, 1.035% (11,165/1,078,822) in 2009, 1.102% (12,029/1,092,067) in 2010, 1.127% (12,806/1,135,156) in 2011, 1.209% (13,985/1,156,326) in 2012 and 1.243% (14,775/1,188,860) in 2013.

### Clinical Outcomes and Costs

The *PCD study sample* among patients with a cancer diagnosed comprised 1,065 individuals. The majority of patients were female (79.8%), and hypertension was a common co-morbid illness, present in 60.5% of the cohort. Obesity was relatively less common (19.0%) (**Table 1**).

**Table 1:** Demographic and clinical characteristics of the PCD study sample as of their index date (the date of first PCD claim) [n = 1,065].

	#	% or SD
**Demographics characteristics as of index date**		
Age in years, Mean (SD)	61.2	13.0
Age in category No. (%)		
0–18	0	0.0%
19–44	98	9.2%
45–64	567	53.2%
65+	400	37.6%
Female, No. (%)	850	79.8%
Race, No. (%) (n = 718)		
Asian	14	1.3%
Black	174	16.3%
Hispanic	59	5.5%
White Non-Hispanic	755	70.9%
Unknown	63	5.9%
**Clinical Characteristics in baseline (12-month pre-device)**		
Obesity, No. (%)	202	19.0%
Diabetes, No. (%)	269	25.3%
Hypertension, No. (%)	644	60.5%
Renal Disease, No. (%)	150	14.1%
Charlson Index, Mean (SD)	4.4	2.4
**Socio-Economic Characteristics in baseline (12-month pre-device)**		
Census region, No. (%)		
Midwest	224	21.0%
Northeast	134	12.6%
South	525.	49.3%.
West	97	9.1%
Other	0	0.0%
Unknown	85	8.0%
Business Line, No. (%)		
Commercial	776	72.9%
Medicare	289	27.1%
Average income, Mean (SD)	$61,840	$26,124
	n = 980	

No., number; PCD, pneumatic compression device; SD, standard deviation.

#### Clinical Outcomes

Health outcomes for cancer-associated lymphedema patients treated with a PCD are shown in **Table 2**. At baseline, patients with lymphedema suffered relatively frequent hospitalizations, with inpatient care provided to 45% of these patients in the year prior to PCD prescription. Use of a PCD was associated with a significant decline in the rate of hospitalizations (45% to 32%, p<0.0001). While lymphedema-related hospitalizations were infrequent at baseline, the rate was slightly lower during the post-PCD period; this difference was not statistically significant (3% vs. 2%, p = 0.41 cancer cohort).

**Table 2:** Clinical Outcomes in the PCD Study Sample (n = 1,065).

		Baseline (12 Months Pre-Device)		Followup (12 Months Post-Device)		Change (Post - Pre)	P-value for Change (Post-Pre)
**Lymphedema-Related Outcomes**	Patients with Hospitalization, # patients (%)	27	3%	22	2%	0%	0.4111
	Number of Hospitalizations, Mean (SD)	0.03	0.23	0.03	0.27	0.00	0.9192
	Patients with Outpatient Hospital Visits, # patients (%)	497	47%	346	32%	−14%	<.0001
	Number of Outpatient Hospital Visits, Mean (SD)	2.76	6.00	1.96	6.38	−0.80	0.0001
	Patients with Cellulitis Dx., # patients (%)	222	21%	77	7%	−14%	<.0001
	Courses of Physical Therapy, Mean (SD)	0.48	1.05	0.41	1.03	−0.07	0.0172
	Use of Physical Therapy, # patients (%)	313	29%	221	21%	−9%	<.0001
**Non-Lymphedema-Related Outcomes**	Patients with Hospitalization, # patients (%)	482	45%	345	32%	−13%	<.0001
	Number of Hospitalizations, Mean (SD)	0.89	1.43	0.67	1.50	−0.22	<.0001
	Patients with Outpatient Hospital Visits, # patients (%)	990	93%	942	88%	−5%	<.0001
	Number of Outpatient Hospital Visits, Mean (SD)	12.49	15.11	9.73	15.84	−2.76	<.0001
	Patients with Cellulitis Diagnosis # patients (%)	295	28%	201	19%	−9%	<.0001
	Courses of Physical Therapy, Mean (SD)	0.55	1.28	0.48	1.13	−0.08	0.0216
	Use of Physical Therapy, # patients (%)	297	28%	270	25%	−3%	0.0934
**All Outcomes**	Patients with Hospitalization,	482	45%	345	32%	−13%	<.0001
	# unique patients (%)						
	Number of Hospitalizations, Mean (SD)	0.92	1.48	0.7	1.57	−0.22	<.0001
	Patients with Outpatient Hospital Visits, # unique patients (%)	1,009	95%	959	90%	−5%	<.0001
	Number of Outpatient Hospital Visits, Mean (SD)	15.25	16.89	11.69	17.83	−3.56	<.0001
	Patients with Cellulitis Dx., # unique patients (%)	295	28%	237	22%	−5%	0.0003
	Courses of Physical Therapy, Mean (SD)	1.04	1.63	0.88	1.52	−0.15	0.0007
	Use of Physical Therapy, # unique patients (%)	529	50%	434	41%	−9%	<.0001

Notes:

Lymphedema-related: ICD-9 code of (457.0,457.1,757.0) in primary or secondary position of claim. All other claims grouped with Non-Lymphedema-Related.

Number of hospitalizations, Outpatient Hospital Visits, and courses of PT are for patients with 1 or more of the visit type.

P-values: Continuous variables tested pre minus post with paired t-test. Binary variables tested with McNemar's test.

Reductions were also observed in the proportion of patients with outpatient hospital visits (95% to 90%, p<0.0001). The percentage of patients with a lymphedema-related clinic visit decreased from 47% in the baseline period to 32% in the post-PCD period (p<0.0001). The proportion of patients with cellulitis also declined in the post-PCD period (28% to 22%, p<0.0003). Finally, the proportion of patients using physical therapy declined (50% to 41%, p<0.0001). These reductions were primarily driven by a reduction in the use of lymphedema-related physical therapy (29% to 21%, p<0.0001); there was no statistically significant change in use of non-lymphedema-related physical therapy.

#### Health Care Costs

Aggregate total costs per patient in the baseline 12-month period were $62,190 for the individuals with cancer-related lymphedema (**Table 3**). Provision of inpatient services constituted a large contribution to the total costs ($15,458) as well as the hospital outpatient services ($21,222) and office visits ($15,278).

**Table 3:** Health Care Costs of the PCD Study Sample (n = 1,065).

		Cancer-Related Patients	Followup (12 Months Post-Device)	Change (Post - Pre)	P-value for Change (Post-Pre)
**Lymphedema-Related Costs**	DME Other	$17	$678	$661	<.0001
	Home Health	$174	$246	$72	0.0491
	Emergency	$39	$36	−$3	0.7972
	Inpatient	$421	$230	−$191	0.2754
	Outpatient Hospital	$1,155	$697	−$458	<.0001
	Outpatient PT costs	$276	$135	−$142	<.0001
	All Other Outpatient Costs	$879	$563	−$316	<.0001
	Office	$415	$475	$60	0.4341
	Lab	$4	$1	−$3	0.3818
	Other Service Location	$17	$20	$3	0.7672
	Pharmacy	$0	$0	$0	.
	Total Cost less DME Other	$2,226	$1,705	−$521	0.0129
	**Total Cost**	$2,243	$2,383	$140	0.5046
**Non-Lymphedema-Related Costs**	DME Other	$298	$334	$36	0.4399
	Home Health	$1,135	$983	−$152	0.3142
	Emergency	$1,463	$1,591	$128	0.4084
	Inpatient	$15,037	$15,687	$651	0.712
	Outpatient Hospital	$20,066	$14,141	−$5,925	<.0001
	Outpatient PT costs	$136	$106	−$30	0.1547
	All Other Outpatient Costs	$19,930	$14,035	−$5,895	<.0001
	Office	$14,863	$9,272	−$5,591	<.0001
	Lab	$864	$535	−$329	0.0001
	Other Service Location	$399	$329	−$70	0.6663
	Pharmacy	$5,822	$5,600	−$222	0.3996
	Total Cost less DME Other	$59,649	$48,139	−$11,510	<.0001
	**Total Cost**	$59,947	$48,473	−$11,473	<.0001
**Total Costs**	DME Other	$315	$1,012	$698	<.0001
	Home Health	$1,309	$1,229	−$80	0.6054
	Emergency	$1,502	$1,627	$126	0.4193
	Inpatient	$15,458	$15,918	$460	0.7961
	Outpatient Hospital	$21,222	$14,838	−$6,383	<.0001
	Outpatient PT costs	$413	$241	−$172	<.0001
	All Other Outpatient Costs	$20,809	$14,597	−$6,211	<.0001
	Office	$15,278	$9,747	−$5,531	<.0001
	Lab	$868	$536	−$333	0.0001
	Other Service Location	$416	$349	−$67	0.6801
	Pharmacy	$5,822	$5,600	−$222	0.3996
	Aggregate Total Cost less DME Other	$61,875	$49,845	−$12,031	<.0001
	**Aggregate Total Cost**	$62,190	$50,857	−$11,333	<.0001

P-values: Continuous variables tested pre minus post with paired t-test.

Binary variables tested with McNemar's test.

The majority of the total costs in the baseline period were non-lymphedema-related: lymphedema-related costs accounted for only 4% of the total costs ($2,243). The largest components of lymphedema-related costs were outpatient services ($1,155), office visits ($415), inpatient services ($421) and home health care ($174).

Use of a PCD was associated with a remarkable decrease in total costs of 18% ($62,190 to $50,857, p<0.0001) in the 12 months after device prescription. The largest cost decreases were achieved by a diminution of office visit costs by 36% (p<0.0001), and outpatient hospital costs by 30% (p<0.0001). Inpatient costs largely remained stable ($15,458 to 15,918, p = 0.7961).

Costs directly attributable to lymphedema care remained stable ($2,243 to $2,383, p = 0.5046). Reductions were observed in lymphedema-related costs for outpatient physical therapy ($276 to $135, p<0.0001) and other outpatient services ($879 to $563, p<0.0001).

## Discussion

Despite its clinical implications for affected individuals, and its high impact on both quality-of-life and functional independence, lymphedema remains a largely overlooked vascular condition in many health care delivery settings. This may reflect the fact that the clinical research community has not yet adequately characterized the population-based prevalence of lymphedema, its health outcomes, and the costs and benefits of lymphedema therapy. Limited public awareness of the condition, and, among practitioners, inadequate appreciation for the natural history and effective treatment options, can be considered to be the consequences of this knowledge gap [27].

Our study provides the first reliable estimate of the population-based prevalence of lymphedema in cancer- related settings. We demonstrate that lymphedema affects a significant and increasing segment of the cancer population. Our study population comprised of both the commercially insured and Medicare Managed Care enrollees from a large US managed care health insurer. For the lymphedema prevalence estimates, our study included an estimated 35 million enrollees of which slightly over 1 million had a primary cancer diagnosis per year. Based on estimates of the US Census and the Current Population Survey, there were about 165 million privately insured, and 13 million Medicare Managed Care enrollees in the US in 2012 [28], suggesting a cancer population of about 5.8 million individuals and among them, a lymphedema burden of 70,000 patients per year among the comparable population nationally. Inclusion of the Medicare Fee-For-Service enrollees (29 million), the Medicaid and other public insurance enrollees (54 million), and the uninsured as of 2012 (48 million) [28], increases the estimated annual prevalence estimates for lymphedema among approximately 10 million patients with primary cancer diagnosis to more than 121,000.

Prior studies have demonstrated the reduction of symptoms and partial-to-complete restoration of functional status with therapeutic interventions for lymphedema [17]–[19]; however it has not previously been demonstrated that current lymphedema treatments fundamentally alter clinical outcomes. This study was designed to focus on the therapeutic intervention of PCD use, because such devices are increasingly used, are accessible to patients when other lymphedema therapies (e.g., manual lymphatic drainage) are not, and serve as a model from which to estimate the benefits of a representative form of compressive therapy. Our data demonstrate, for the first time, the healthcare utilization patterns among cancer-associated lymphedema patients, and the association of PCD use with improvement in several clinical outcomes of interest. We observed that the use of a PCD was associated with a favorable impact on the rate of hospitalizations, clinic visits, cellulitis, and the use of physical therapy, all of which represent important clinical and therapeutic endpoints that reflect patient health status and quality of life.

The claims dataset we relied upon allows a longitudinal observation of the health economic burden associated with clinical care of the cancer population with lymphedema. Baseline health economic costs prior to acquisition of the PCD were high, with the majority of these costs found to be non-lymphedema-related. These treatment-related costs are accrued in diverse care settings, spanning home health care to inpatient sites. Our economic analysis is based on the measurement of direct costs alone, and does not include the costs of transportation to care sites, time lost from work, or losses due to the limited mobility and function that are known to be associated with lymphedema. Nonetheless, we found that economic costs decreased significantly in the year after PCD acquisition. Of note is the fact the largest component of cost reduction was reflected in outpatient care, which reduced by 36%, suggesting that there was a reduction in outpatient visits. Hospitalization costs remained relatively stable, suggesting, inferentially, that PCD was effective in reducing the health care costs of mild-to-moderate lymphedema cases, but not those of the more severely ill individuals.

### Limitations

Our study has several potential limitations. Claims data are derived from populations of insured individuals and thus provide only an estimate of incidence and prevalence; they do not yield information about lymphedema patients without health insurance. The attributional fidelity of coding for lymphedema when associated medical care is provided is not known, and thus we may be predisposed to significantly underestimate the actual lymphedema-related costs. Another potential limitation of this study is the potential for overlap of the pre- and post-device observations with the natural history of the cancer care, namely, a greater magnitude of cancer morbidity and cost in the earlier phases of observation. This is an unavoidable limitation of an observational study of this type, which lacks a control population. Finally, as in any observational study, this analysis is limited by the potential influence of confounding. We do not have a comparable control group of patients who were untreated, limiting the ability to draw causal conclusions.

## Conclusions

Our study provides the first population-based estimate of national lymphedema prevalence among cancer survivors. This is almost certainly an underestimate, as not all cases are coded and evaluable in an administrative dataset. Nonetheless the estimates reflect a prevalence comparable to, or exceeding, many other disease states that have been more thoroughly investigated and supported by the medical community. These data also demonstrate that health care costs associated with a new lymphedema diagnosis are high, but that a suitable therapeutic intervention, such as PCD use, might mitigate the economic impact. The prior lack of health outcomes data of this nature has contributed to the under-recognition of lymphedema, and may have limited patient access to lymphedema treatment resources. This is particularly relevant in the cancer-survivor population, where the advent of lymphedema has a particularly profound impact on quality-of-life and general health.

The potential public health implications of these findings are substantial. The aging American population can be predicted to contribute to continued increases in disease prevalence over time. The availability of effective home care therapies is likely to become increasingly important with downward pressures on reimbursement. Further prospective studies are needed to more precisely define prevalence attributes for this disease and to quantify the nature of the potential benefit of therapeutic interventions for lymphedema.
